# EHMTI-0036. Gammacore device for treatment of migraine attack: preliminary report

**DOI:** 10.1186/1129-2377-15-S1-G12

**Published:** 2014-09-18

**Authors:** L Grazzi, S Usai, G Bussone

**Affiliations:** 1 Headache Center, Neurological Institute C. Besta, Milano, Italy

## Introduction

despite the several options for treatment of migraine attack , sometimes pain episodes remain unresolved and often patients ask for non pharmacological methods for aborting attack which can be a valid alternative to the use of medications.

## Aim

The application of a simple and non invasive VNS device (gammacore) for treatment of migraine attack.

## Method

Thirthy patients, aged 18-65, suffering from migraine without aura according with HIS criteria, (5-9 attacks per month), were included in this open-label , single arm, multiple attack study.

Patients, after a specific educational training, treated from 3 to 6 migraine episodes by the portable VNS device. Treatment consisted of one, 90 seconds doses delivered to the right cervical branch of the vagus nerve.

## Results

Ninety six migraine attacks were treated globally by a single shot application.

Ninety six attack were treated : 43 attacks were resolved completely within 30 minutes (44.8%); for 42 (43.7%) attacks the application did not show any benefit in the first 2 hours so patients recurred to rescue medication; in 11 (11.4%) attacks the result was uncertain: no resolution of attack, only a moderate relief of pain.

No adverse evens were recorded

## Conclusions

The results suggest that VNS may be an effective and well tolerated treatment for migraine attack in a good percentage of cases, with one shot modality.

Randomized controlled studies are needed also to fix up the adequate dosage to apply the device for the resolution of pain.

No conflict of interest.

